# Development of Pluoronic nanoparticles of fluorocoxib A for endoscopic fluorescence imaging of colonic adenomas

**DOI:** 10.1117/1.JBO.28.4.040501

**Published:** 2023-04-20

**Authors:** Md. Jashim Uddin, Hiroaki Niitsu, Robert J. Coffey, Lawrence J. Marnett

**Affiliations:** a Vanderbilt University School of Medicine, Department of Biochemistry, Nashville, Tennessee, United States; b Vanderbilt University Medical Center, Department of Medicine, Nashville, Tennessee, United States; c Vanderbilt University Medical Center, Division of Gastroenterology, Hepatology and Nutrition, Department of Medicine, Nashville, Tennessee, United States; d Vanderbilt University, Department of Chemistry, Nashville, Tennessee, United States; e Vanderbilt University School of Medicine, Department of Pharmacology, Nashville, Tennessee, United States

**Keywords:** Fluorocoxib A, Pluronic^®^ nanoparticles, cyclooxygenase-2, colorectal adenomas, fluorescence colonoscopy

## Abstract

**Significance:**

Current white light colonoscopy suffers from many limitations that allow 22% to 32% of preneoplastic lesions to remain undetected. This high number of false negatives contributes to the appearance of interval malignancies, defined as neoplasms diagnosed between screening colonoscopies at a rate of 2% to 6%.

**Aim:**

The shortcomings of today’s white light-based colorectal cancer screening are addressed by colonoscopic fluorescence imaging of preneoplastic lesions using targeted fluorescent agents to enhance contrast between the lesion and the surrounding normal colonic epithelium.

**Approach:**

We describe the development of Pluronic^®^ nanoparticles of fluorocoxib A (FA), a fluorescent cyclooxygenase-2 (COX-2) inhibitor that enables targeted imaging of inflammation and cancer in numerous animal models, for endoscopic florescence imaging of colonic adenomas.

**Results:**

We formulated FA, a fluorescent COX-2 inhibitor, or fluorocoxib negative control (FNC), a nontargeted fluorophore and a negative control for FA, in micellar nanoparticles of FDA approved Pluronic tri-block co-polymer using a bulk solvent evaporation method. This afforded FA-loaded micellar nanoparticles (FA-NPs) or FNC-loaded micellar nanoparticles (FNC-NPs) with the hydrodynamic diameters ($D_{h}$) of 45.7±2.5  nm and 44.9±3.8  nm and the zeta potentials (ζ) of −1.47±0.3  mV and −1.64±0.5  mV, respectively. We intravenously injected B6;129 mice bearing colonic adenomas induced by azoxymethane and dextran-sodium sulfate with FA-loaded Pluronic nanoparticles (FA-NPs). The diffusion-mediated local FA release and its binding to COX-2 enzyme allowed for clear detection of adenomas with high signal-to-noise ratios. The COX-2 targeted delivery and tumor retention were validated by negligible tumor fluorescence detected upon colonoscopic imaging of adenoma-bearing mice injected with Pluronic nanoparticles of FNC or of animals predosed with the COX-2 inhibitor, celecoxib, followed by intravenous dosing of FA-NPs.

**Conclusions:**

These results demonstrate that the formulation of FA in Pluronic nanoparticles overcomes a significant hurdle to its clinical development for early detection of colorectal neoplasms by fluorescence endoscopy.

## 1 Introduction

**Fig. 1:**
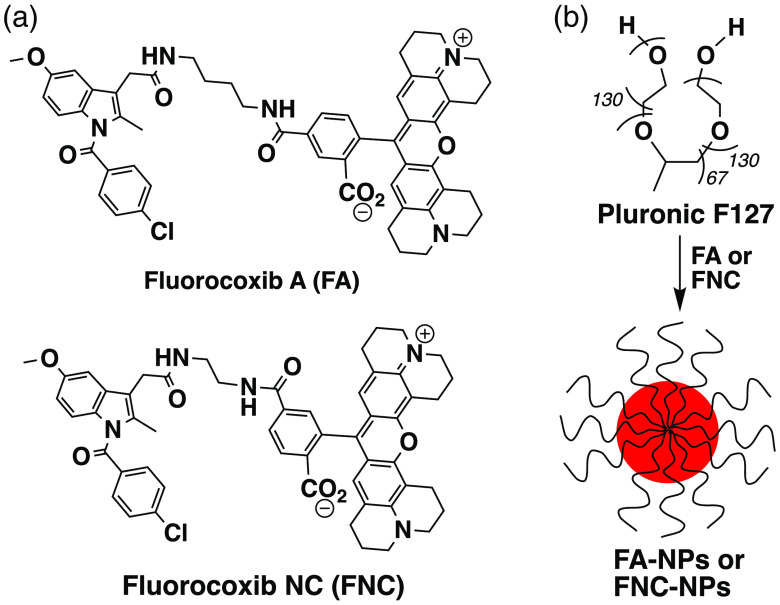
(a) Chemical structure of FA and FNC. (b) Pluronic F127 polymeric micellar nanoparticle formulation of FA or FNC in ${\mathrm{CHCl}}_{3}$ and DPBS following gentle stirring at 25°C for 16 h in the dark.

The enzyme, cyclooxygenase-2 (COX-2), is overexpressed in a wide variety of malignant and premalignant neoplasms, contributing to both progression and metastasis.^1^^–^^3^ COX-2 expression has been particularly well-documented in colorectal cancer (CRC) with an average of 86% of malignancies exhibiting detectable levels of COX-2 in multiple studies.^4^^–^^8^ Among benign and premalignant colonic lesions, COX-2 expression levels range from 12.5% in hyperplastic polyps to 90% in adenomas.^4^^–^^11^ In addition, COX-2 expression increases as the lesion progresses, suggesting that it can serve as a biomarker of early CRC. Thus COX-2 is a promising target for the development of imaging agents to enable the detection of premalignant and malignant lesions of the colon. To this end, we developed fluorocoxib A (FA) [Fig. 1(a)], a potent and selective fluorescent COX-2 inhibitor comprising the nonsteroidal anti-inflammatory drug indomethacin conjugated to the fluorophore, carboxy-X-rhodamine (ROX), through a four-carbon linker.^12^^–^^18^ We hypothesize that FA-mediated fluorescence colonoscopy will enable COX-2-targeted visualization of adenomas of the colon. In earlier preclinical studies, FA enabled visualization of a wide range of COX-2-expressing neoplasms, including intestinal polyps in ${\mathrm{Apc}}^{\text{Min}}$ mice, 1483 human head and neck squamous cell carcinoma tumor xenografts in nude mice,^15^ nonmelanoma skin cancer in mice,^14^ and head-and-neck cancer and colorectal and bladder cancer in dogs.^12^^,^^13^ These results suggest that the clinical development of FA has the potential to substantially improve the early detection of neoplasms including CRC. However, development has been hampered by FA’s lack of aqueous solubility. To address this issue, we now report the Pluronic^®^ formulation of FA and describe its utility in COX-2-targeted colonoscopic optical imaging of azoxymethane/dextran sodium sulfate (AOM/DSS)-induced adenomas of the colon in B6;129 mice.

## 2 Materials and Methods

Previously, FA has been solubilized using solvent systems that are not approved by the FDA for clinical applications.^15^^,^^19^ Thus we selected Pluronic, an FDA-approved organic nonionic triblock copolymer, consisting of a central hydrophobic block of polypropylene glycol (PPG) edged by two hydrophilic blocks of polyethylene glycol (PEG), for the formulation of FA. Pluronic polymer self-assembles into micellar nanoparticles in aqueous systems, enabling solubilization of hydrophobic organic compounds, such as FA, by encapsulating them inside the hydrophobic core surrounded by the hydrated corona.

### 2.1 Pluronic F127 Polymeric Micellar Nanoparticles

We encapsulated FA into Pluronic F127 (${\mathrm{PEG}}_{101}\text{-}{\mathrm{PPG}}_{56}\text{-}{\mathrm{PEG}}_{101}$, also known as Poloxamer 407) polymeric micelles by a bulk solvent evaporation method.^18^ In this method, FA and Pluronic F127 polymer were first dissolved individually in chloroform. Then the FA (25 mL, 20 mg/mL) solution was added to the Pluronic (25 mL, 200 mg/mL) solution, and the resultant solution was added dropwise to 1 mL of Dulbecco’s phosphate buffered saline (DPBS pH 7.0 to 7.3, without calcium and magnesium) at 25°C with gentle stirring. The binary system was stirred for 16 h at 25°C in the dark, during which time the chloroform was evaporated. Sterile centrifugation of the formulation afforded FA-loaded Pluronic micellar nanoparticles (FA-NPs) ready to administer for colonoscopic fluorescence imaging of adenomas of the colon. As a negative control, we used fluorocoxib negative control [FNC, Fig. 1(a)] for fluorescence colonoscopy because it lacks the ability to inhibit COX-2 in both purified enzyme and cell-based assays.^15^ The FNC was encapsulated in Pluronic F127 [Fig. 1(b)] micelles using a bulk solvent evaporation method identical to that used for the FA formulation described above. This afforded FNC-loaded Pluronic micellar nanoparticles (FNC-NPs).

### 2.2 Concentration of FA in Pluronic Micellar Nanoparticles Solution

We measured the concentration of FA-encapsulated Pluronic micellar nanoparticles (FA-NPs) solution by adding dimethylformamide (DMF, 0.1 mL) to FA-NPs solution (0.1 mL). We stirred the mixture for 16 h at 25°C to release FA from the nanoparticles. This was performed in triplicate. Then we measured the fluorescence intensity of each test sample at 605 nm emission (excitation at 581 nm) on a TECAN Infinite M1000Pro Micro-Plate Reader, from which we calculated the concentration of FA in each test sample by comparing the fluorescence intensity values with a standard curve of FA ($\lambda_{\mathrm{ex}}$: 581 nm, $\lambda_{\mathrm{em}}$: 605 nm) dissolved in 50/50 DMF/DPBS. This afforded the FA concentration to be 0.231 mg/mL in FA-NPs solution.

### 2.3 Photophysical and Physicochemical Properties of Pluronic F127 Micellar Nanoparticles

We determined the photophysical properties of Pluronic F127 micellar nanoparticles on a Spex 1681 Fluorolog Spectrofluorometer containing a 450-W Xenon Arc Lamp. Using a previously described procedure,^20^ we recorded the excitation and emission spectra of both FA-NPs and FNC-NPs at a 0.05 AU/cm optical density measured in 10×4  mm cuvettes (FA-NPs, $\lambda_{\mathrm{ex}}$ at 581 nm and $\lambda_{\mathrm{em}}$ at 605 nm and FNC-NPs, $\lambda_{\mathrm{ex}}$ at 581 nm and $\lambda_{\mathrm{em}}$ at 606 nm). We used a Malvern Zetasizer Nano-ZS Instrument, equipped with a 4-mW Helium Neon Laser operating at 632.8 nm to measure the hydrodynamic diameter ($D_{h}$) and zeta potential (ζ) of the nanoparticles. In both cases, the samples were prepared by passing the micelles dissolved in 1 mL of DPBS without calcium and magnesium (pH 7.3) through a 0.45-μm syringe filter.

### 2.4 Animal Model of Colorectal Adenoma

We used a colorectal adenoma mouse model^21^ in a mixed C57BL/6J and 129S1/SvlmJ background, named B6;129 mice. We housed and treated all mice according to a protocol approved by the Institutional Animal Care and Use Committee at the Vanderbilt University Medical Center. We treated 8-week-old wild-type B6;129 mice with two 10 mg/kg intraperitoneal doses of azoxymethane (AOM; Sigma-Aldrich, St. Louis, Missouri, United States) in phosphate-buffered saline (pH 7.2) in 2 consecutive days (1 dose/day) followed by free drinking of sterile deionized water containing 2% dextran sulfate sodium (DSS; #DS1044, Gojira Fine Chemicals, Ohio, United States) for 5 days. After completion of DSS administration, the mice were allowed to drink distilled water for 8 to 10 weeks. In this model, colorectal adenomas begin to appear at the distal colon 8 weeks after AOM injection, which is a substantially shorter latency time for tumor development than that of the classical colonic tumorigenesis model induced by AOM alone.^22^ Morphologically these mouse adenomas are similar to human colonic tumors.^23^^,^^24^

### 2.5 Veterinary Endoscopy System

We used a Karl Storz-Endoskope System (Karl Storz, Germany) for fluorescence colonoscopy of B6;129 mice bearing colonic adenomas. Mice were anesthetized with isoflurane (Piramal Critical Care, Bethlehem, Pennsylvania, United States). The endoscopy system was configured with a rigid telescope. The dimension of the telescope was 1.9  mm×10  cm with a 30-deg field of amplification. The telescope was connected to a Tricam camera and a modified D-light source with RFP/mCherry filter sets for simultaneous fluorescence and white-light colonoscopic *in vivo* imaging.

### 2.6 In Vivo Endoscopic Fluorescence Imaging and Validation

B6;129 mice (4 animals/group) bearing AOM/DSS-induced colorectal adenomas were administered with FA-NPs (0.5 mg/kg) or FNC-NPs (0.5 mg/kg) via tail vein injection. In COX-2 blocking control experiments, mice received FA-NPs (0.5 mg/kg) 1 h after an intraperitoneal injection of celecoxib (10 mg/kg) to block binding of FA to the COX-2 active site. Endoscopic fluorescence imaging experiments were performed at 1-h postinjection of FA-NPs, celecoxib/FA-NPs or FNC-NPs using a Karl Storz-Endoskope System. We recorded videos and captured static images of colonic adenomas and surrounding normal colon epithelium during the fluorescence colonoscopy.

### 2.7 Quantification of Fluorescence Intensity

Following endoscopic imaging of all four groups of animals (n=4 animals/group), the captured static images were analyzed. It should be noted that, among the test or control group of animals, each mouse possessed one tumor (n=4 tumors/group). We created regions of interest (ROIs) within the colonic adenomas or surrounding normal colon epithelium and measured the fluorescence intensity within these ROIs using ImageJ software.

### 2.8 Determination of Signal-to-Background Ratio

We measured the signal-to-background ratio (SBR) by dividing the mean fluorescence intensity of colonic adenomas ($6.72\times 10^{9}$, n=4 tumors) with the mean fluorescence intensity of surrounding normal colon epithelium ($2.31\times 10^{8}$, n=4 normal tissues) within the respective ROIs measured in photons/s using ImageJ software.

### 2.9 Statistical Methods

For statistical analyses of acquired data, we used student’s t-test to compare fluorescence signal intensities between colonic tumors with surrounding normal colon epithelium. We set the statistical significance at P≤0.05, where the data were used as the arithmetic mean and standard error within the size of the samples (n).

## 3 Results and Discussion

We formulated and determined the photophysical properties of FA-NPs and FNC-NPs in aqueous solutions that resulted in complete retention of excitation and emission maxima of FA and FNC. FNC is an analog of FA with a tether between the indomethacin and ROX moieties that is 2-carbons long rather than the 4-carbon tether in FA. This shorter tether renders FNC unable to inhibit COX-2. In addition, we determined the physicochemical properties including hydrodynamic diameter and zeta potentials. The data showed that the hydrodynamic diameter ($D_{h}$) of FA-NPs and FNC-NPs were 45.7±2.5  nm and 44.9±3.8  nm, respectively, and the zeta potentials (ζ) were −1.47±0.3  mV and −1.64±0.5  mV, respectively. These results suggest that both FA-NPs and FNC-NPs exhibit properties reported to be favorable for tissue penetration and cellular internalization in living animals.^18^

We used a colorectal adenoma mouse model^21^ in B6;129 mice by treating them with AOM followed by DSS. In this model, adenomas are typically observed at 8 weeks after AOM administration. The tumors appeared to be predominantly oval with an average length of 1 to 2 mm and width of 0.5 to 1.0 mm.

**Fig. 2:**
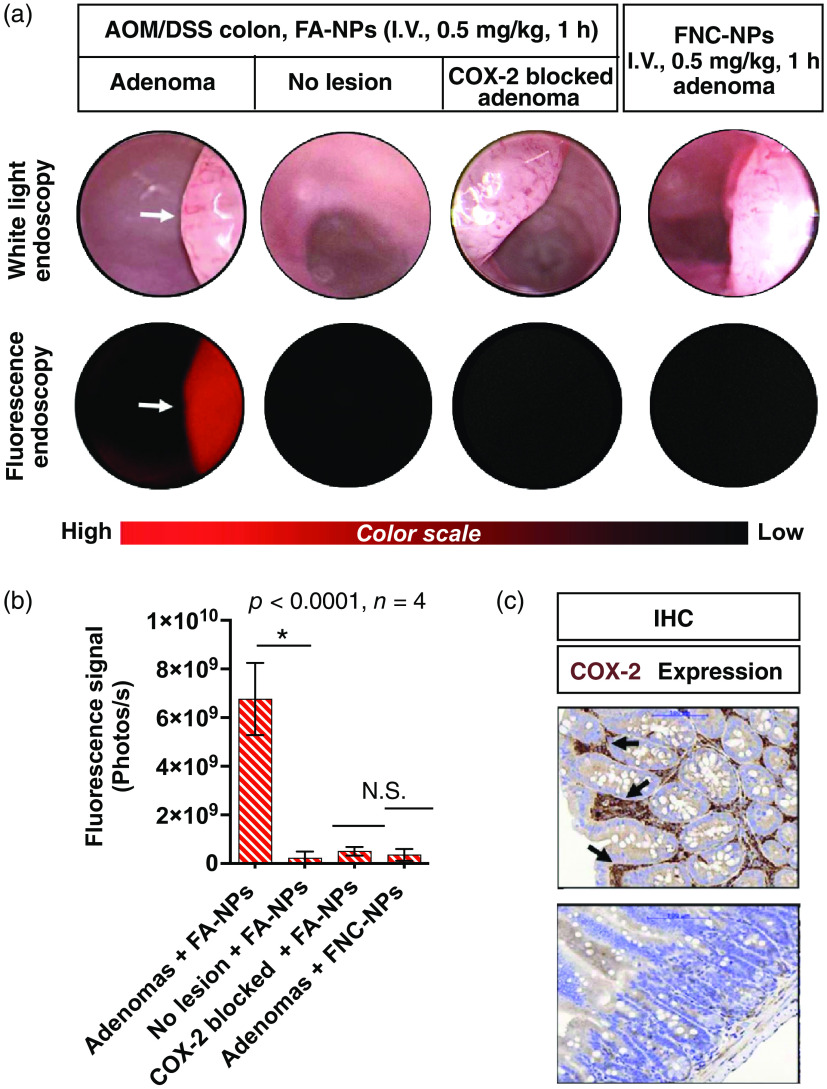
(a) Representative white light and corresponding fluorescence colonoscopy images (static) of B6;129 mice bearing either colorectal adenomas or normal colon injected with Pluronic micellar nanoparticles of fluorocoxib A (FA-NPs, 0.5 mg/kg) or Pluronic micellar nanoparticles of fluorocoxib negative control (FNC-NPs, 0.5 mg/kg) via tail vein injections. In the COX-2 blocking control experiment, tumor bearing B6;129 mice received FA-NPs (0.5 mg/kg) 1 h after an intraperitoneal injection of celecoxib (10 mg/kg) to block binding of FA to the COX-2 active site. (b) ImageJ software was used for quantification of fluorescence intensity at the ROI of colonic adenomas versus adjacent normal colon epithelium (N.S., not significant). (c) COX-2 immunohistochemical staining of well-differentiated adenoma and adjacent normal colon (Video 1, MOV, 31.9 MB [URL: https://doi.org/10.1117/1.JBO.28.4.040501.s1]).

FA-NP or FNC-NP was injected intravenously and 1 h later mice were lightly anesthetized with 2% isoflurane, and fluorescence colonoscopy was performed using a Karl Storz Veterinary Colonoscope. The delivery of FA-NPs followed by the release of FA resulted in a high adenoma fluorescence compared with that of the surrounding normal colon tissues [Fig. 2(a), Figs. S1 and S2 in the Supplementary Material]. The SBRs were >29 (n=4, p<0.0001). In contrast, minimal fluorescence of adenomas was detected at 1 h postdosing of FNC-NPs in tumor-bearing mice. Similarly, minimal fluorescence was observed in animals treated with the COX-2 inhibitor, celecoxib, before administration of FA-NP [Fig. 2(a)]. We used ImageJ software for postacquisition image analysis [Fig. 2(b)]. The expression of COX-2 in the tumor tissues was confirmed by immunohistological analysis [Fig. 2(c)]. Histologically, the tumors were well-differentiated adenomas. Of interest, the COX-2 immunoreactivity was confined to the stroma of the adenoma, and staining was not observed in the normal colon. These results confirm that the FA delivery and tumor retention occurred by a COX-2-dependent mechanism.

White light colonoscopy has revolutionized our understanding and ability to characterize colorectal neoplasms in appreciable detail. It also provides a highly effective means to screen, diagnose, and treat preneoplastic and neoplastic colonic lesions. It has been hypothesized that fluorescence colonoscopy using a specific probe targeted to a CRC biomarker can improve the detection of preneoplastic colonic lesions. Past efforts to test this hypothesis have included the development of optical imaging agents targeted to a range of CRC biomarkers.^25^ Currently, peptide-based compounds^26^ and antibody-based agents^27^ are still at the preclinical stages. The present studies with targeted nanoparticles set the framework for endoscopic molecular imaging of COX-2 as a biomarker of CRC in animal models and will facilitate the development of this nanotechnology for early detection colorectal neoplasm in patients.

## 4 Conclusions

In this letter, we described FA-NPs, Pluronic polymer-based micellar nanoparticles of FA, and demonstrated their COX-2-dependent delivery of FA to colonic neoplasms, thereby enabling FA-mediated colonoscopic optical imaging of adenomas in AOM/DSS-treated B6;129 mice. The COX-2-dependent delivery of FA within the colorectal adenomas was confirmed by the presence of very low fluorescence signal in adenomas from animals injected with nontargeted FNC-NPs or animals treated with celecoxib prior to injection with FA-NPs. This study establishes a framework for clinical development/deployment of FA-NPs for colonoscopic fluorescence imaging of COX-2 as a biomarker for the early detection of colorectal cancer.
