# Supplementary material for: Development of Pluoronic nanoparticles of fluorocoxib A for endoscopic fluorescence imaging of colonic adenomas
Source: J Biomed Opt. 2023 Apr 20;28(4):040501. doi: 10.1117/1.JBO.28.4.040501 (PMC10118138; doi:10.1117/1.JBO.28.4.040501)
Supplement: Supplementary file 1 [file JBO_028_040501_SD001.pdf]

Supporting Information

Development of Pluoronic Nanoparticles of Fluorocoxib A for Endoscopic Fluorescence Imaging of Colonic Adenomas

Md. Jashim Uddin,[a,*] Hiroaki Niitsu,[b] Robert J. Coffey,[c] and Lawrence J. Marnett,[d,*]

[a]Department of Biochemistry, Vanderbilt University, Nashville Tennessee 37232 USA

[b]Department of Medicine, Vanderbilt University Medical Center, Nashville Tennessee 37232 USA

[c]Department of Medicine, Division of Gastroenterology, Hepatology and Nutrition, Vanderbilt University Medical Center, Nashville Tennessee 37232 USA

[d]Departments of Biochemistry, Chemistry and Pharmacology, Vanderbilt University School of Medicine, Nashville Tennessee 37232 USA

List of Contents

Figure 1S. White light and fluorescence colonoscopy…………………..Pg#2

Figure 2S. Video of a fluorescence colonoscopy………………………..pg#3

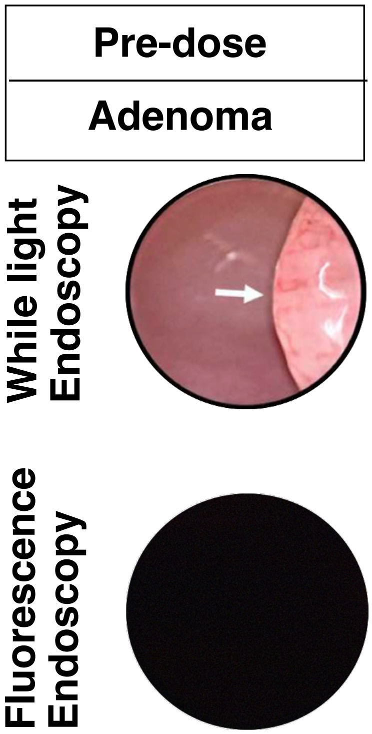

Figure 1S. White light and fluorescence colonoscopy of a B6;129 mice bearing an AOM/DSS–induced adenoma before administration of FA-NPs probe.

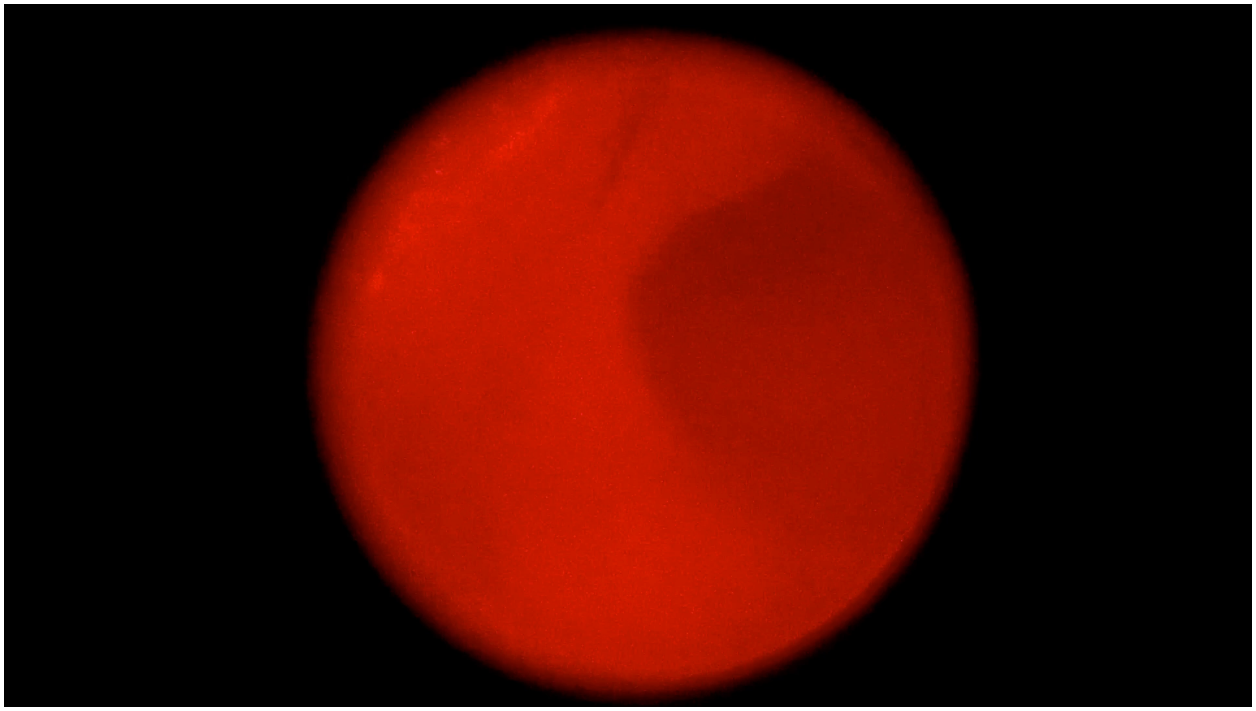

Figure 2S. A video clip of fluorescence colonoscopy of a B6;129 mice with AOM/DSS–induced adenoma at 1 h post-administration of FA-NPs probe (i.v., 0.5 mg/kg dose).
